# Drug delivery systems based on biocompatible imino-chitosan hydrogels for local anticancer therapy

**DOI:** 10.1080/10717544.2018.1466937

**Published:** 2018-05-03

**Authors:** Daniela Ailincai, Liliana Tartau Mititelu, Luminita Marin

**Affiliations:** a Petru Poni Institute of Macromolecular Chemistry, Grigore Ghica Voda Alley, Iasi, Romania;; b Grigore T. Popa University of Medicine and Pharmacy, Iasi, Romania

**Keywords:** Hydrogels, drug delivery, enzymatic degradability, biocompatibility, anticancer activity

## Abstract

A series of drug delivery systems were prepared by chitosan hydrogelation with citral in the presence of an antineoplastic drug: 5-fluorouracil. The dynamic covalent chemistry of the imine linkage allowed the obtaining of supramolecular tridimensional architectures in which the drug has been homogenously dispersed. Fourier-transform infrared spectroscopy (FTIR), wide-angle X-ray diffraction (WXRD) and polarized light microscopy (POM) measurements were used in order to follow the hydrogelation and drug encapsulation processes. The ability of the prepared systems to release the drug has been investigated by UV–Vis spectroscopy using a calibration curve and by fitting the results with different mathematic models. To mimic the behavior of the hydrogel matrix in bio-environmental conditions in view of applications, their enzymatic degradability was monitored in the presence of lysozyme. The *in vivo* side effects of the systems, in terms of their influence on the blood elements, biochemical and immune parameters were monitored on white Swiss mice by intraperitoneal administration of the injectable obtained hydrogels. All the characteristics of the obtained systems, such as micro-porous morphology, uniform drug encapsulation, enzymatic degradability, lack of side effects, other than the one of the drug itself, along with their ability to release the drug in a sustained manner proved that these material meet the requirements for the development of drug delivery systems, making them suitable for being applied in intraperitoneal chemotherapy.

## 1. Introduction

Hydrogels represent a class of materials with unique physical properties which make them suitable for a large range of applications in the biomedical field (Hoffman, 2002; Enas, 2015). Therefore, hydrogels have been widely used as scaffolds in tissue engineering (Lee & Mooney, 2001; Sherbiny & Magdi, 2013) or restorative medicine (Brandon et al., 2009; Yang et al., 2014), in diagnostics (Buengera et al., 2014), as membranes for biomolecules separation (Jung & Kinam Park, 1998) or as matrix for drugs encapsulation, transportation and release (Graham & McNeill, 1984; Hoare & Kohane, 2008; Jianyu & Mooney, 2016). The main characteristics of hydrogels such as high water content, high porosity, high deformability and moldability along with the versatility of these properties which can be easily modified usually by changing the crosslinking degree, represent important advantages sustaining their applications in biomedicine (Nabanita et al., 2011; Jalalvandi, 2017).

In particular, hydrogels based on chitosan represent an important part among this class of soft materials because they have a high level of biocompatibility and therefore applicability, conferred on one side by the high amount of water and on the other side by the nature and intrinsic properties of chitosan: stability, susceptibility to enzymatic degradation, nontoxicity, and so on (Ahmadi et al., 2015; del Valle et al., 2017). More than this, the hydrogels based on polysaccharides, including chitosan, present the advantages of being similar to the external extracellular matrix, from both compositionally and mechanically points of view (Sherbiny & Magdi, 2013). Therefore, there are many hydrogels based on chitosan which have been used in the design of drug delivery systems for different routes of administration: oral (George & Abraham, 2006), ocular (Kumar et al., 2012; Chenga et al., 2016), vaginal (Knuth et al., 1993) or buccal (Xu et al., 2015) and for different diseases, including cancer, which occupies four from the top 10 causes to death in the world (World Health Organization, 2002). The development of anticancer drug delivery systems based on hydrogels attracted a lot of interest in the scientific world due to their ability to increase the drug half-life and versatility in terms of releasing rate and doses (Gary et al., 2013). Moreover, the fact that hydrogels based drug delivery systems may be applied locally makes them suitable for the curative or palliative treatment of serious gynecological and gastrointestinal malignancies, like peritoneal carcinomatosis (Coccolini et al., 2013). Taking in consideration that the peritoneum is the membrane which surrounds the abdominal organs, the peritoneal carcinomatosis is associated with a poor prognosis for the patients (Marz & Piso, 2015). Even if there are plenty of antineoplastic agents on the market used for the amelioration of peritoneal carcinomatosis (Lu et al., 2010), their systemic delivery generates many side effects due to the spreading of the chemotherapeutic agents through the whole body (De Angelis, 2008), leading to many and unbearable consequences for the patient: hair loss, nausea, damage to mouth, teeth and mucosa, diarrhea or constipation or muscle pain (Aslam et al., 2014). That is why, lately appeared the so called local chemotherapy which consists in applying the drugs at the site of the tumor with or without inducing a hyperthermic effect (Spiliotis et al., 2016).

In this context, our objective was the obtaining and characterization of an anticancer drug delivery system (CFU system) based on a novel biocompatible hydrogel originating from natural sources (Marin et al., 2017) and an antineoplastic drug: 5-fluorouracil (5FU). The hydrogel, designed and synthesized in our laboratory, presents the advantage of thixotropy that allows its injection in the peritoneal cavity, assuring the premises of a prolonged release of the encapsulated drug with a maximum effect in the peritoneum and minimum side effects in the rest of the body. As method of loading, has been chosen the hydrogelation in the presence of the drug, thus increasing the possibility to obtain more homogenous systems leading to a prolonged release.

## 2. Material and methods

### 2.1. Material

Citral (95%), low molecular weight chitosan (217 kDa, DD (deacetylation degree): 85%), 5-fluorouracil, lysozyme from chicken white egg and phosphate buffer solution have been purchased from Aldrich (Germany) and used as received.

### 2.2. Methods

The structural characterization of the prepared systems was done by Fourier-transform infrared (FTIR) Spectroscopy, using a FT-IR Bruker Vertex 70 Spectrophotometer (Ettligen, Germany), by ATR technique on xerogels. The spectra have been processed with OPUS 6.5 software (Ettligen, Germany).

Wide-angle X-ray diffraction (WXRD) was performed on xerogels pellets, using a Bruker D8 Avance diffractometer (Bruker, Germany) with the Ni-filtered Cu-Kα radiation (λ = 0.1541 nm). The working conditions were 36 kV and 30 mA and data were handled by the FullProf 2000 program (Bruker, Germany). All the diffractograms were registered in the range of 2–45 (2θ°). The xerogels were previously compacted using a manual Hydraulic Press, applying a pressure of 10 N/m^2^.

The xerogels morphology was investigated with a field-emission scanning electron microscope (SEM) EDAX Quanta 200 (Eindhoven, Germany) at accelerated electron energy of 20 keV.

The gelation time was determined when visually the reaction mixture was transformed from viscous to elastic/rubbery state.

Polarized light microscopy (POM) of the hydrogels was performed with an Olympus BH-2 polarized light microscope (Hamburg, Germany), on thin samples placed between two lamellae.

The xerogels have been obtained by lyophilization from the corresponding hydrogels, using a Labconco FreeZone Freeze Dry System (Canada) (FreeZoner2.5 Liter Freeze Dry Systems) equipment for 24 h at −50 °C and 0.04 mbar.

The drug release kinetics has been monitored by UV–Vis spectroscopy using a Perkin Elmer Lambda 35 UV-Vis spectrophotometer (Paris, France). The calibration curve for the drug has been previously traced using the absorption maximum from its spectrum, at 265 nm. The release kinetics from the developed drug delivery systems has been monitored by registering the absorbance at 265 nm from the supernatant in which the release was done, after which the concentration was calculated using the Beer-Lambert law.

The *in vitro* enzymatic degradation of the reference hydrogels has been evaluated by measuring the mass loss of the hydrogels in lysozyme buffer solution in comparison with the mass loss of the same amount of hydrogel in PBS. With this aim, pieces of the hydrogel CC4, the one which allowed a sustained release of the encapsulated drug, with a mass of 0.4 g have been added to 20 mL of lysozyme solution 1 g/L and kept at 37 °C (Chandrawati, 2016). At certain time intervals the pieces were took out, washed with double distilled water to remove the salts and submitted to analysis. The mass loss determination was calculated using Equation (1) as follows:

(1) $$W_{loss}=\frac{W_{0}-W_{t}}{W_{0}}\times 100$$

where, *W*_loss_ = the weight loss of the hydrogel, *W*_0_ = the initial weight of the lyophilized hydrogel, *W*_t_= the weight of the lyophilized hydrogel after was immersed in PBS or lysozyme solution a certain time interval.

In order to evaluate the morphological changes after the enzymatic degradation, the pieces of hydrogels which were kept several days in lysozyme solution have been frozen in liquid nitrogen, lyophilized and analyzed by scanning electron microscopy. For comparison, were analyzed also the hydrogels kept in PBS and used as reference.

The *in vivo* side effects of the drug delivery systems were evaluated by assessing their impact on the blood elements, the serum biochemical tests and immune parameters, after intraperitoneal injection of hydrogels in white Swiss mice (Foltz, 1999; Botham, 2004). The mice weighted between 25 and 30 g and were kept at a temperature of 22–24 °C, 55–65% relative humidity, in a constant 12-h light/12-h dark cycle (with a light period between: 7 am and 7 pm), with free access to water and standard food except the period of the studies. Before the experiments, the animals were placed on a raised wire mesh, under a clear Plexiglas box and allowed 2 h to familiarize to the laboratory environment. After a quarantine period of 1 week, the animals were randomly distributed into five groups of six mice each, treated intraperitoneally, in unique administration, according to the Table 1. 24 h and 7 days, respectively, after administration, two blood samples of 0.3 mL each have been collected from the ophthalmic vein of the mice anesthetized with enflurane, and serum was separated by centrifugation at 2500 rpm, for 15 min, in a refrigerated centrifuge. The count of blood elements and the liver enzymes activity were examined using the hematologic Analyzer 5 DIFF model BF-5180 (Budapest, Hungary).

**Table 1.** Samples and doses of intraperitoneally administration.

Group	Administrated substance	mg 5FU drug	mg chitosan
I	Distilled water 0.1 mL/10 g body weight (bw)	–	–
II	Chitosan solution	–	60
III	5FU	2.05	–
IV	CFU3	2.05	60
V	CFU4	2.05	60

At these two moments of the experiment, the following parameters: red blood cells (RBC), white blood cells (WBC) and platelets number, glutamic oxaloacetic transaminase (GOT), glutamic pyruvic transaminase (GPT) and lactic dehydrogenase (LDH) levels were determined (De Jong et al., 2008; Wolf et al., 2012).

In the seventh day, serum opsonic capacity (OC) (using *Staphylococcus aureus 94* cultures) was also measured. At the end of the experiment animals were sacrificed, after general anesthesia with enflurane, and the peritoneal macrophages were removed from the intact peritoneal cavity with 10 ml HANKS solution thermostatated at 37 °C. Samples were centrifuged (at 1000 rpm for 10 min), brought into contact with *Staphylococcus aureus 94* cultures, incubated for 48 h at 37 °C and re-inseminated on culture media. The following immune parameters: phagocytic capacity (PC) and bactericidal capacity (BC) of peritoneal macrophages, were assessed. These parameters belongs from a battery test used to evaluate immunologic influence of different substances in laboratory animals (Lupuşoru, 2001; Peacman, 2011).

The investigations have been performed in the interval between 8 am and 1 pm in order to prevent the chrono-biologic interferences.

Data were expressed as mean values ± standard deviation (SD) and statistically analyzed using SPSS program (IBM Corporation, New York, USA), variant 17.00 for Windows and one-way analysis of variance (ANOVA) method. The values of *p* (probability) bellow .05 were considered to be statistically significant, comparing to control group.

The study protocol was conducted in accordance with the University of Medicine and Pharmacy ‘Grigore T. Popa’−Iasi Ethic Committee on Research, in compliance with the EU Directive 2010/63/EU regarding the protection of animals used for experimental studies.

For ethical reasons, the duration of the tests was kept as short as possible, each animal was used once only, and sacrificed at the end of the experiment (*AVMA Guidelines on Euthanasia*, 2007) (***Protocole d'amendement à la convention européenne sur la protection des animaux vertébrés utilisés à des fins expérimentales ou à d'autres fins scientifiques. Strasbourg; 22 June 1998).

### 2.3. Experimental protocol for the obtaining of the drug delivery systems and the reference hydrogels

Two series of four samples each have been prepared: a first one by encapsulating the same amount of an anticancer drug: 5-fluorouracil into four different hydrogels obtained from chitosan and citral, varying the molar ratio of amino groups to aldehyde, from 4:1 to 1:1 (Table 2). These systems will be further noted with CFU, followed by a number representing the molar ratio between NH_2_ groups of chitosan and CHO group of citral. For example, CFU2 represents the hydrogel obtained by a molar ratio of 2:1 between NH_2_ and CHO groups. The second series consists in reference hydrogels, without drug, based only on chitosan and citral in the same molar ratios as previously mentioned (Table 2). The reference hydrogels will be further noted CC, followed by a number representing the molar ratio between the reactive groups. Therefore, the CC2 sample was obtained by reacting chitosan with citral in a molar ratio of their functionalities equal to 2.

**Table 2.** The codes and the NH_2_/CHO molar ratios used for hydrogels obtaining.

Sample code	m_chitosan_ (mg)	V_acetic acid aq. sol._ (mL)	m_citral_ (mg)	V_ethanol_ (mL)	V_water_ (µL)	m_5FU_ (mg)	Gelation time (min)	m_dried substance_ (mg)	m_xerogel_ (mg)	Yield (%)
CFU1	60	2	48.5	4.85	200	39	0	147.5	146.8	99.5
CFU2	60	2	24.03	2.4	200	39	2	123.03	122.85	99.8
CFU3	60	2	16	1.6	200	39	10	115	114.2	99.3
CFU4	60	2	12	1.2	200	39	1440	111	110.35	99.4
CC1	60	2	48.5	4.65	200	–	5	108.5	107.85	99.4
CC2	60	2	24.03	2.2	200	–	20	84.03	83.01	98.7
CC3	60	2	16	1.4	200	–	70	76	75	98.6
CC4	60	2	12	1	200	–	1440	72	71.2	98.8

The drug–hydrogels conjugates (CFU systems) were prepared by an *in situ* hydrogelation procedure of chitosan with citral in the presence of 5FU, as follows. A solution of 5FU and citral in a mixture of water and ethanol was added to a 3% chitosan solution acetic acid solution in water (0.7%), under vigorous magnetic stirring, at 55 °C (Table 2). The volume of ethanol was calculated in order to achieve the final concentration of citral of 1%. The amount of drug has been established to be 39 mg, because experimentally was observed that amounts higher than that prevented gel formation. During the adding, the system became a yellow viscous liquid that further transformed into a semisolid. The time interval necessary for hydrogelation depended on the molar ratio of the functional groups (Table 2). The hydrogels were transparent, with no macroscopic particles inside, indicating a fine dispersion of the drug into the hydrogel mass. As an example, the synthesis of CFU2 (for which the ratio between NH_2_:CHO is 2:1), was the following one: to a solution of 3% chitosan obtained by dissolving 60 mg chitosan (0.303 mmol glucosamine units) in 2 mL of acetic acid solution in water (0.7%), a solution of 24.03 mg citral (0.151 mmol) and 39 mg of 5FU into a mixture of 2.2 mL ethanol and 200 µL water, was added at 55 °C.

For the hydrogels used as reference (CC systems), the experimental protocol was similar, except the use of 5FU drug (Table 2).

In order to characterize the resulted systems from the morphological and supramolecular points of view, all the CC and the CFU samples were lyophilized, leading to the corresponding xerogels. By weighting the reagents and the resulted xerogels, it was established that no weight loss occurred during lyophilization (Table 2).

## 3. Results and discussions

**Scheme 1.**
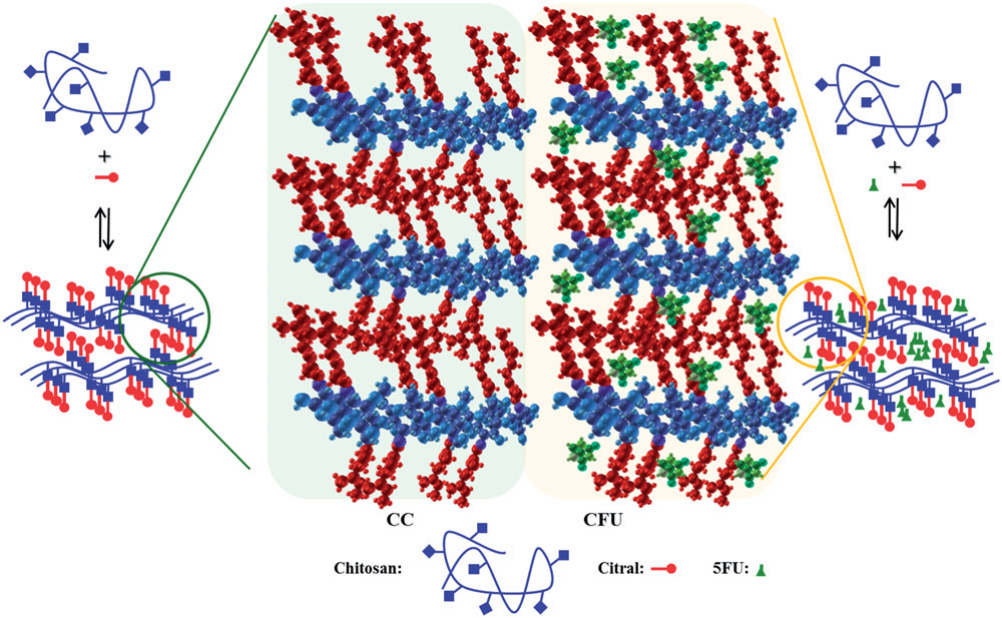
The obtaining of CC and CFU systems.

A series of four drug delivery systems has been obtained by encapsulating an anticancer drug (5FU) into four different hydrogels based on chitosan and citral with different crosslinking ratios (Scheme 1).

Hydrogels based on chitosan (2% w/v) and citral, have already proved *in vivo* biocompatibility (Marin et al., 2017) and that is why we evaluated further the possibility of these systems to act as a matrix for drug delivery. Unfortunately, the obtaining of hydrogels using the same experimental protocol, more precisely at a concentration of chitosan of 2% was not possible, the systems remaining only slightly viscous even after 10 days, without forming a gel. That is why, the experimental protocol was changed and the concentration of the chitosan solution was increased, up to the value which allowed the gel formation, 3%.

### 3.1. Structural analysis by FTIR

**Figure 1.**
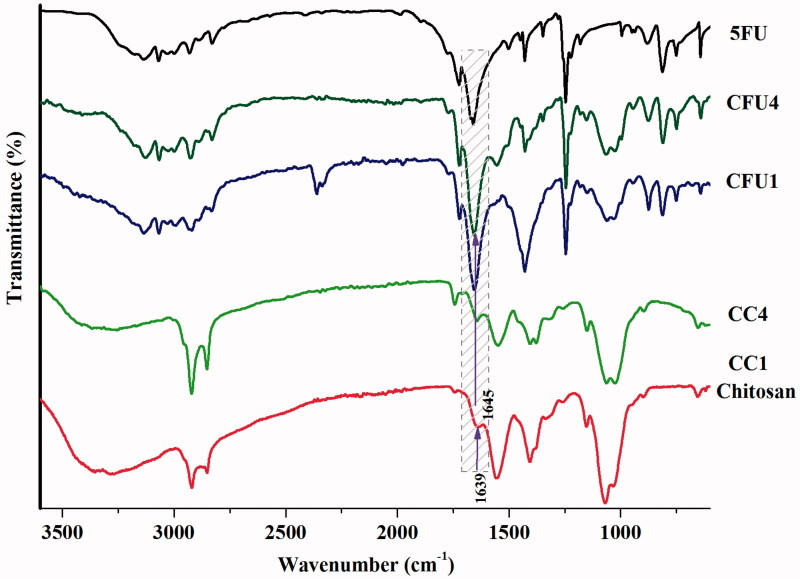
FTIR spectra of chitosan, 5 FU and representative CC and CFU samples.

Taking into consideration that the drug encapsulation occurred simultaneously with the imination reaction, exists the risk that because of steric hindrance the citral molecules to not be able to reach the active amine groups on chitosan backbone, impeding imination. From this reason, the xerogels containing the drug have been structurally characterized by FTIR spectroscopy. For comparison, the FTIR spectrum of the 5FU has been also recorded. The FTIR spectrum of the drug presents a band at 812 cm^1^ which corresponds to the stretching vibration outside the plan of the C–H bond, bands at 1243 cm^−1^ and at 1672 and 1657 cm^−1^ characteristic to the stretching vibrations of the C=C bond and the group vibration of C=O, respectively, and also a broad band at 3120 cm^−1^ characteristic to the stretching vibrations of the NH bond (Patel et al., 2011). The formation of imine linkages between chitosan and citral in the CFU systems has been demonstrated by the appearance of an absorption band around 1645 cm^−1^ in the finger print region of the FTIR spectra (Figure 1) and the disappearance of the band corresponding to the vibrations of the aldehyde group (1675 cm^−1^) from citral and also the decreasing in intensity of the band corresponding to the amine group of chitosan (1556 cm^−1^), indicating their consumption in the acid condensation reaction. Even if the highlighting of the formation of the imine linkage in CFU systems is hampered by the fact that, the drug presents a band in the same spectral region in which appeared the band characteristic to the imine, some differences in this spectral region were observed. Therefore, the drug presents a broad band with two absorption maxima at 1672 and 1657 cm^−1^, while in the xerogels spectra the band is shifted to lower wavenumbers and it is formed from multiple overlapped bands: the two maxima of the drug and the band at 1645 cm^−1^, corresponding to the group vibrations of the imine linkage. Moreover, the deconvolution on the band in discussion evidences the individual bands at 1645, 1672 and 1657 cm^−1^ (Supplementary Figure S1).

On the other side, the grafting of citral on chitosan backbone led to different morphological changes, indicated by the significant modifications observed in the 1500–1300 and 1100–1000 cm^−1^ spectral region.

### 3.2. Wide-angle X-ray diffraction

X-Ray diffraction has been used in order to appreciate the state of the dispersed drug and also its influence on the supramolecular architecturing of the hydrogels. As has been already proven, the hydrogelation of chitosan with citral is driven by the hydrophilic/hydrophobic segregation of the dynamic imine amphiphiles which form, due to the antagonistic nature of the two components (hydrophilic chitosan and hydrophobic citral), ordered clusters which act as network nodes (Marin et al., 2017).

**Figure 2.**
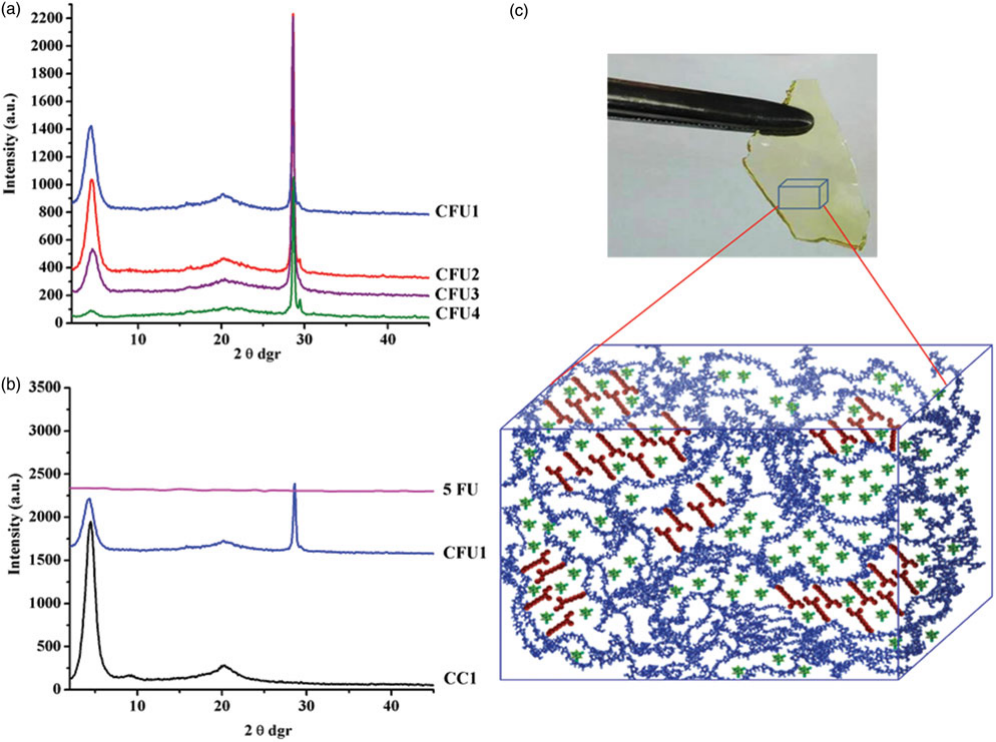
X-Ray diffractograms of (a) CFU systems and (b) CC1, CFU1 and 5FU and (c) hydrogel picture and the representation of the three-dimensional supramolecular architecture of the CFU systems.

As well-known in the literature, 5FU showed multiple reflection bands in line with its supramolecular triclinic crystallization system (Supplementary Figure S2) (Fallon, 1973). As can be seen in Figure 2(b) the reflection bans of the 5FU are intense, especially the bands from 28, 29, 32 and 39 2θ° attributed to the intermolecular hydrogen bonds which form between the oxygen and nitrogen atoms within the same layer (Supplementary Figure S2). The 5FU diffraction pattern presents another two peaks, at 21 and 16 2θ°, corresponding to inter-layer distance between the atoms situated in two different layers, from the supramolecular architecture (Supplementary Figure S2).

As can be seen in Figure 2, the X-ray diffractograms of the hydrogels encapsulating the 5FU drug show reflections which belong to both components. Comparing with the reference hydrogels, in the diffractograms of the CFU xerogels, it could be observed the presence of the same three peaks at 4.4, 9 and 20.3°, respectively. This confirmed the same supramolecular architecture of the CFU systems as of the reference hydrogels. The different crosslinking degrees of the CFU1–CFU4 samples reflected in different intensities of the peaks indicating the formation of a different number of ordered clusters. The intensity of the three peaks is lower in the diffractograms of CFUs than those of the corresponding CCs, pointing for ordered clusters of smaller dimensions, in agreement with the drug presence, which impedes the hydrophilic/hydrophobic segregation (Figure 2). Comparing with the diffractogram of the 5FU drug, in the diffractograms of the CFU xerogels were encountered peaks of lower intensities at 16 and 29.4 2θ°, according with the presence of the drug as smaller crystals. Moreover, the most intense reflection is at 28.6 2θ°, corresponding to the dominance of the hydrogen bonds between the 5FU molecules forming layers, while the band at 16 2θ° corresponding to the inter-layer distances almost disappeared. This pattern suggests likely bi-dimensional architectures of the drug into the hydrogel, result of stronger interfacial forces between drug and hydrogel matrix than inter-layer forces within the drug.

Therefore, from the X-Ray data, it can be concluded that exists a mutual influence of both components: the drug makes more difficult the hydrophilic/hydrophobic segregation while the viscous medium hampers the drug crystallization.

### 3.3. Morphological characterization by SEM

**Figure 3.**
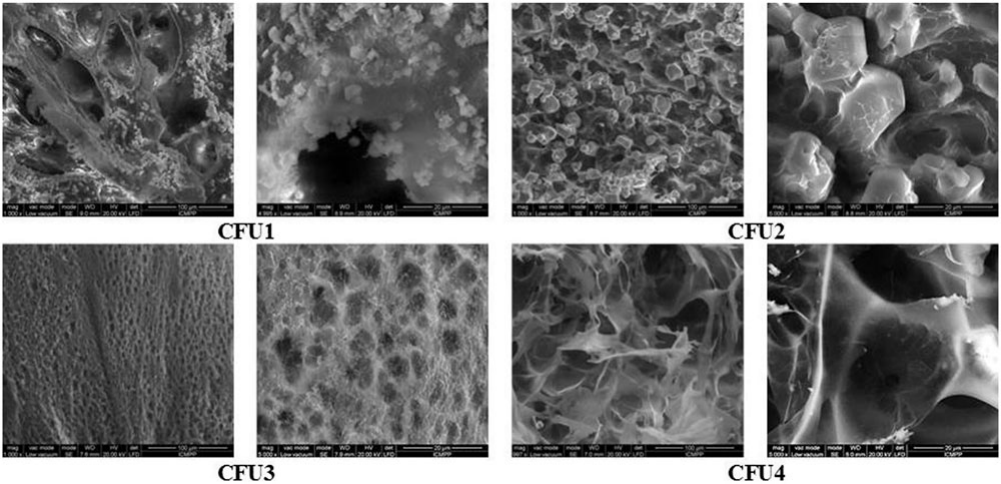
SEM images of the CFU systems.

All hydrogels, regardless the presence or the absence of the drug, presented a porous morphology (Figure 3).

The CFU systems presented the same sponge-like microstructure as the reference hydrogels (Marin et al., 2017), with micrometric pores, depending on the used NH_2_/CHO molar ratio. On the other hand, the higher viscosity of the CFU1 and CFU2 and the rapid gelation do not allow a good dispersion of the drug, part of it growing as big crystals with a mean diameter around 8 µm. In the SEM images of the CFU1 and CFU2 xerogels, were observed microcrystals in both the walls and the pores of the xerogels (Figure 3). The samples CFU3 and CFU4 do not show any visible drug crystals, very probably due to their sub-micrometric size, as was indicated by X-ray measurements. This means that the drug was more intimately dispersed into the hydrogels mass due to the lower viscosity and the higher gelation time of these two systems which allow the drug molecules to disperse and to grow as small crystals.

### 3.4. Polarized optical microscopy

It is known that the polarized optical microscopy is a rapid method to observe the samples ordering. The samples CFU1 and CFU2 show drug crystals dispersed in slide birefringent matrix, while CFU3 and CFU4 show fine granular birefringent texture (Supplementary Figure S3). These observations confirmed on a hand, the SEM data, indicating the forming of smaller drug crystals when the crosslinking degree of the hydrogels was lower and the forming of drug crystals of different size when the crosslinking degree was higher. On the other hand, the birefringence confirms the high ordering of the samples originating from the crystalline drug and also from the ordered clusters of the hydrogels matrix. Moreover, the birefringence of the hydrogels CFU3 and CFU4 is higher than the one of the mass which surrounds the crystals in CFU1 and CFU2, indicating that this birefringence is given besides the hydrogel itself, by the drug which is homogeneously distributed in the hydrogel mass.

### 3.5. The ability of the CFU systems to release the 5FU drug

In order to evaluate the ability of the obtained systems to release the encapsulated 5FU, at first it was obtained a calibration curve of the drug, by UV–Vis spectroscopy, using the absorption maximum from 265 nm (Supplementary Figure S4), according to a method described in the literature (Cojocaru et al., 2012).

Further, pieces of hydrogels containing the same amount of drug have been introduced in PBS, at 37 °C. At different time intervals, from the buffer solution, 2 mL have been extracted which were analyzed by UV–Vis spectroscopy.

**Figure 4.**
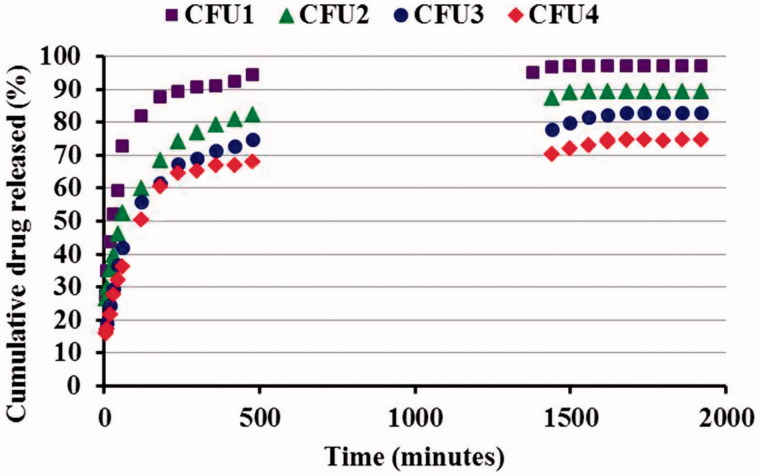
Release kinetics of 5FU from the CFU systems.

As it could be observed (Figure 4), the hydrogels released the encapsulated drug differently, depending mainly on the crosslinking degree. Thus, the CFU1 hydrogel released the drug very fast, after 4 h being released ∼90% from the entire amount of 5FU, while in the same time interval the CFU2 sample released 75%, the CFU3 70%, while the CFU4 only 65%. This fact can be explained if we take into consideration that in the case of the CFU1 and CFU2 samples, the drug is not very well-dispersed into the hydrogels mass, forming even micrometric crystals, as was demonstrated by SEM and POM images. In the case of the CFU3 and CFU4, characterized by a lower release rate, the drug is homogeneously distributed as submicrometric crystals into the hydrogels.

Another difference consists in the fact that the CFU1 releases more than 95% from the entire amount of encapsulated drug, while the CFU4 releases only 75%, up to the time of the last determination. All hydrogels presented a burst effect, higher for CFU1 and significantly lower for CFU4, but this is very probably because of the high amount of drug encapsulated in the hydrogels, meaning 35.6% in the case of the sample CFU1 and 26.4% in the case of the sample CFU4.

In order to obtain information regarding the mechanism of release, the kinetic data were fitted using different mathematic models: Korsmeyer-Peppas, Higuchi and Zero Order (Bibek, 2010) (Supplementary Figure S5). The zero-order model was not appropriate for this study, leading to values of the *R*^2^ parameter bellow 0.96. This means that the release of the 5FU from the CFU systems is not controlled by the solubility of the drug, but by another parameter. In order to elucidate it, the kinetic data were further analyzed using the other two mathematic models, mentioned before.

The Korsmeyer-Peppas model was applied, using the following equation:

$$\frac{M_{t}}{M_{\infty }}=kt^{n_{r}}$$

where, *M*_t_/*M*_∞_ represents the amount of the released drug, *M*_t_ and *M*_∞_ are the amounts of released drug at *t* moment and ∞ and *k* is a constant which depends on the used matrix and also on the drug and the *n*_*r*_ exponent gives information about the release mechanism.

From the obtained values for the *R*^2^ for the Kormeyer-Peppas, it could be observed that this model describes quite well the mechanism of the 5FU release from the hydrogels (Supplementary Table S1). Moreover, the values obtained for the n_r_ which is lower than 0.5 indicated a pseudo Fickian release, controlled by the diffusion of the drug from the hydrogel (Bibek, 2010).

Further, the Higuchi model was applied on the kinetics data, using the well-known equation:

$$\frac{M_{t}}{M_{\infty }}=kt^{\frac{1}{2}}$$

From the obtained values of *R*^2^ (>0.99), it could be concluded that this mathematical model describes much better the release of the 5FU from the hydrogels, indicating that the drug is released at first from the superficial layers and after that, when the superficial layers remain almost without drug, it starts to diffuse along the matrix to the superficial layers (Bibek, 2010).

The two mathematic models which led to concordant results, Korsmeyer-Peppas and Higuchi, proved that the release of the 5FU drug from the CFU systems is controlled by the drug diffusion from the superficial layers, the bulk of hydrogel acting as a drug reservoir during the releasing process.

### 3.6. Enzymatic degradability of the matrix: gravimetric analysis and scanning electron microscopy

The biodegradability of a hydrogel is an important parameter which must be taken into consideration when it is used as a matrix for drug delivery systems (Mi, 2005). Therefore, the degradability of the hydrogel CC4 was further investigated in (i) PBS and (ii) lysozyme solution in PBS, at human body temperature (37 °C). Among enzymes, lysozyme presented interest for us because of its abundance in different human’s secretions such as saliva and mucus. The changes have been investigated in terms of mass loss by gravimetric analysis and morphological changes by SEM.

**Figure 5.**
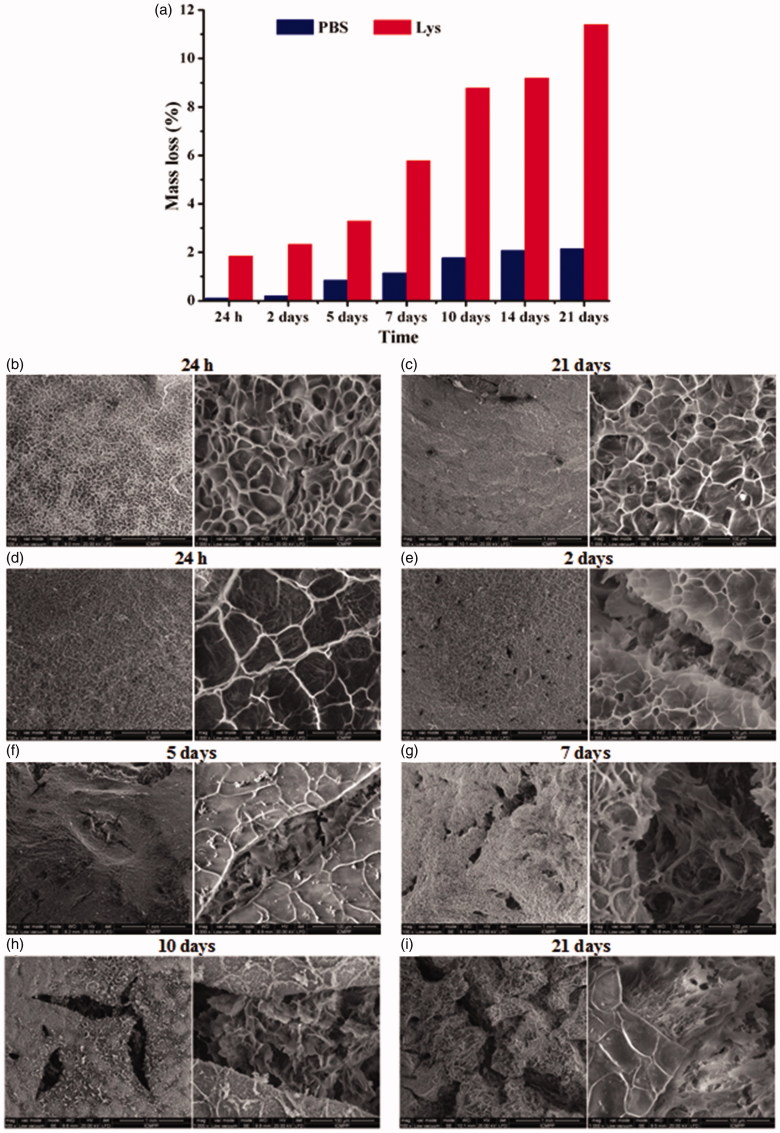
The mass loss in the case of the hydrogel CC4 in PBS (blue) and lysozyme solution (red) at 37 °C and the morphology of the CC4 samples after they were kept different time intervals in (b,c) PBS and (d–i) lysozyme solution.

As expected, the mass loss increases with the increase of time being more evident when lysozyme was used (Figure 5(a)). Remarkable is the fact that the mass loss at 24 and 48 h, respectively, is insignificant in PBS (∼0.2%), revealing once more the fact that the releasing of the 5FU is a consequence of a diffusion process through the hydrogel network, without its erosion, at least up to 48 h. The mass loss in PBS reaches the maximum value after 21 days, but even after this long time interval, it remains quite low (<3%). This might be surprising because the hydrogel is based on imine linkages, which are known to be reversible in aqueous medium. The explanation can be given by the fact that the hydrogel three-dimensional network is formed by linking the chitosan chains by the ordered clusters of citril-imino-chitosan units which are in fact hydrophobic, limiting the access of water molecules. In lysozyme solution, the mass loss is much higher than in PBS, and these results are obviously an effect of enzyme on the hydrogel structure. Therefore, it is expected that the lysozyme acts on the β-1,4 glycosidic linkages from chitosan backbone, leading to the erosion of the hydrogels. In this manner, the hydrogels structure is destroyed in contact with lysozyme solution, leading to a significant higher mass loss, reaching the maximum after 21 days (>11%).

Further, enzymatic degradation of the hydrogel was compared with the samples held in PBS and evidenced by morphological changes followed by SEM. As can be seen in Figure 5(b,c), for the sample kept in PBS SEM images didn’t show significant morphological changes, while for the enzymatic degradation were obtained different morphologies after a certain time, confirming the degradation demonstrated by the gravimetrical analysis.

With respect to the samples kept in lysozyme, the morphological changes depend on the time in which the hydrogels and lysozyme were kept in contact. Therefore, the morphology of the hydrogel kept for 24 h in lysozyme solution didn’t suffer any significant changes. Moreover, the sample presents almost the same morphology as the reference hydrogel kept in PBS the same time interval. Important morphological changes appeared in the case of the hydrogels which were kept longer than 2 days in lysozyme solution. More precisely, the corresponding samples presented cracks with different shapes and sizes, which are bigger and more numerous with the increase of the time interval in which they were kept in contact with the enzyme. The size of the cracks increases from 50 µm for the sample kept for 2 days, to 280 µm for the sample kept for 21 days in the lysozyme solution, data which correlate very well with the mass loss of the corresponding samples (Figure 5(d–i)).

### 3.7. The evaluation of the side effects after the intraperitoneal administration of the CFU systems by ‘*in vivo*’ tests on mice

The side effects of the 5FU drug are well-known and unfortunately are devastating for the patient (Aslam et al., 2014). Its intravenous or intraperitoneal administration as a solution has immediate effect not only on the cancer cells but on the entire body. Moreover, even if the plasma half-life time of this drug is 10–20 min, its side effects remain in the body for 3–5 days (Richard & Schilsky, 1998) or more. From this point of view, in the development of a 5FU drug delivery system, it is important to not induce a more harmful effect on the body than the drug itself or to assure the release of the drug in a sustained manner, maintaining its side effects, but also its anticancer activity.

Therefore, we further investigated the side effects of CFU3 and CFU4 by intraperitoneal administration on mice and by monitoring the blood elements and some biochemical and immune parameters. For comparison, a chitosan and 5FU solutions have been also intraperitoneally administered and their effect have been investigated.

In the case of chitosan and control (distilled water), the analysis of the blood serum did not present important variations in terms of RBC, WBC and platelets number, at 24 h and 7 days after their administration (Table 3).

**Table 3.** The influence of hydrogels administration, on the number of RBC, WBC and platelets and on the GTP, GOT and LDH activity.

	RBC (×10^6^/mm^3^)	WBC (×10^3^/mm^3^)	Platelets (×10^3^/mL)	GPT (U/mL)	GOT (U/mL)	LDH (U/L)
Control						
24 h	7.25 ± 2.7	8.18 ± 1.7	45.7 ± 1.9	85.2 ± 3.1	339.29 ± 37.21	318 ± 5.7
7 days	7.43 ± 3.3	8.24 ± 1.9	47.8 ± 2.5	87.4 ± 2.3	341.47 ± 40.33	324 ± 4.9
Chitosan						
24 h	7.31 ± 1.2	8.15 ± 2.3	44.5 ± 2.3	87.8 ± 3.3	340.38 ± 36.39	311 ± 5.5
7 days	7.46 ± 2.5	8.22 ± 2.7	49.3 ± 1.7	89.9 ± 1.9	343.51 ± 39.45	319 ± 6.7
5FU						
24 h	9.34 ± 1.9**	5.88 ± 1.5*	51.3 ± 1.5	97.1 ± 2.5	344.26 ± 41.27	145 ± 6.5*
7 days	9.22 ± 3.2**	5.63 ± 2.3*	53.4 ± 2.7	97.9 ± 1.7	347.53 ± 37.51	140 ± 4.3*
CFU3						
24 h	9.35 ± 2.4**	5.82 ± 1.5*	55.6 ± 2,2	98.3 ± 3,5	343.17 ± 43.43	139 ± 5.5*
7 days	9.29 ± 2.7**	5.75 ± 2.4*	58.5 ± 1.9	98.7 ± 2.3	349.45 ± 38.29	135 ± 5.4*
CFU4						
24 h	9.33 ± 3.3**	5.76 ± 2.2*	53.9 ± 2.5	97.4 ± 1.7	345.39 ± 34.17	141 ± 4.2*
7 days	9.25 ± 2.3**	5.68 ± 1.9*	56.7 ± 1.7	98.2 ± 2.5	347.53 ± 38.25	137 ± 5.3*

Values were expressed as mean ± SD.

*p < .05; ** *p* < .01 versus control for six mice in a group.

On contrary, the use of CFU3 and CFU4, as well as, the use of 5FU, were associated with an increase in the number of RBC, respectively a decrease in the number of WBC and platelets after 24 h and 7 days, respectively, in the experiment. A positive observation for the potential applications, especially of the hydrogel CFU4, is the fact that the modification of the tested parameters is not higher than the one of the 5FU solution, indicating that the use of this hydrogel as a drug delivery matrix do not induce any supplementary cytotoxic effect. Furthermore, it is important that the CFU system, which was proved to act as a drug reservoir, without inducing a more harmful effect to the body than the 5FU itself, it is able to locally release the drug in a sustained manner. Moreover, even if there are some modifications in the number of RBC and WBC after the administration of CFU3 and CFU4, the achieved values persist in the normal range registered for Swiss mice (Santos et al., 2016). The decrease of platelets number, associated with the modification on the rheological properties and blood coagulation (Baerlocher et al., 1997), consisted in similar values in the case of the 5FU and the CFU systems, even if 5FU has been administrated in a dose, while a sustained release of the drug took place in the case of CFU.

Even if one of the more severe problems usually associated with chemotherapy is liver failure (Wijaya et al., 2012), 5FU is one of the few antineoplastic substances, which were reported to not damage the liver (El-Sayyad et al., 2009). Therefore, it is important to check if the drug remains liver safe when it is sustained released from the hydrogels. That is why, further were measured GOT, GPT and LDH activity, which are important biomarkers related to the liver functionality. The obtained results indicate that there are no substantial dissimilarities between groups treated with chitosan, 5FU, CFU3, CFU4 and control group, at 24 h and 7 days after their intraperitoneal injection (Table 3). However, a slight increase of the serum liver enzymes levels has been observed, but it is statistically insignificant comparing with the Control group.

The results of immunological tests are presented in Supplementary Table S2. As can be easily observed, the values of the determined immune parameters (CO, PC and BC) corresponding to the mice treated with CFU3 and CFU4 samples, are almost the same with the values of the control, indicating that the studied systems are not perceived by the body immune system as being unsafe.

Summarizing, the intraperitoneal administration of the hydrogels CFU3 and CFU4 resulted in similar blood parameters variations and biochemical modifications compared with 5FU solution in mice, but maintaining them in the normal ranges previously reported for healthy Swiss mice. Moreover, the treatment with the CFU systems did not noticeably influence the mice immune reactivity, comparing with the distilled water-treated group.

Moreover, if we take into consideration the low half-time (10–20 min) of the 5FU drug, and its prolonged side effects (>5 days), the obtaining of similar harm, but cumulated with a sustained release of the drug from the hydrogel network points to a better balance between negative and positive effects, indicating these systems as appropriate for being used in local drug delivery.

## 4. Conclusions

A series of four drug delivery systems have been obtained by the hydrogelation of chitosan and citral by dynamic covalent chemistry, in different molar ratios in the presence of an antineoplastic agent: 5FU, a drug largely used in cancer treatment. The hydrogels proved to have a microporous morphology, with pores characterized by a quite low dimensional polydispersity, which represents an advantage by giving a homogenous response in the biological environment. POM and SEM techniques revealed a homogenous drug encapsulation as sub-micrometric crystals for the two hydrogels with lower crosslinking degree and a larger dimensional polydispersity with crystals up to 10 μm, for the others. The release kinetics, followed by UV–Vis and the fitting on the KP and H mathematic models, revealed a diffusion controlled release mechanism starting from the superficial layers, with a prolonged release for the hydrogel CFU4 for which up to 1920 min was released only 65% from the encapsulated drug. The matrix hydrogels proved to be susceptible to enzymatic degradation, reaching 11% mass loss after 21 days, in the presence of lysozyme. The evaluation of the side effects of the systems compared to the pure drug, revealed similar side effects but with a prolonged release acting as a drug reservoir.

These results encourage the development of such drug delivery systems for the local delivery of antineoplastic agents.

## Supplementary Material

Supplementary_DD_rev.docx
