# Supplementary material for: Drug delivery systems based on biocompatible imino-chitosan hydrogels for local anticancer therapy
Source: Drug Deliv. 2018 May 3;25(1):1080–90. doi: 10.1080/10717544.2018.1466937 (PMC6058698; doi:10.1080/10717544.2018.1466937)
Supplement: Supplementary_DD_rev.docx [file IDRD_A_1466937_SM2760.docx]

Supplementary information

for

**Drug delivery systems based on imino-chitosan biocompatible hydrogels for local anticancer therapy**


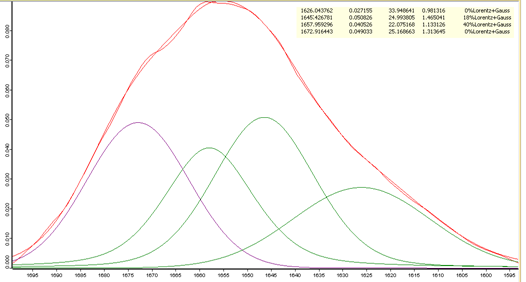


**Figure 1s.** Deconvolution for the CFU2 xerogel


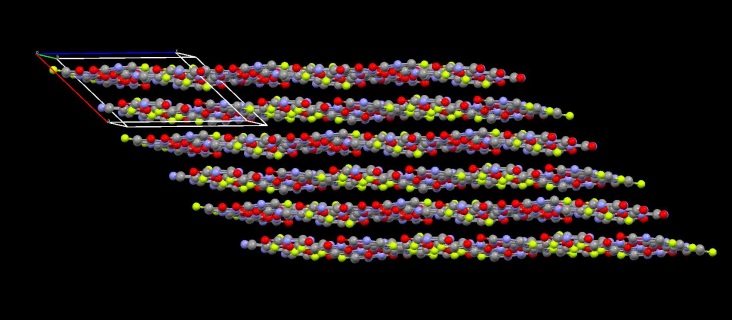


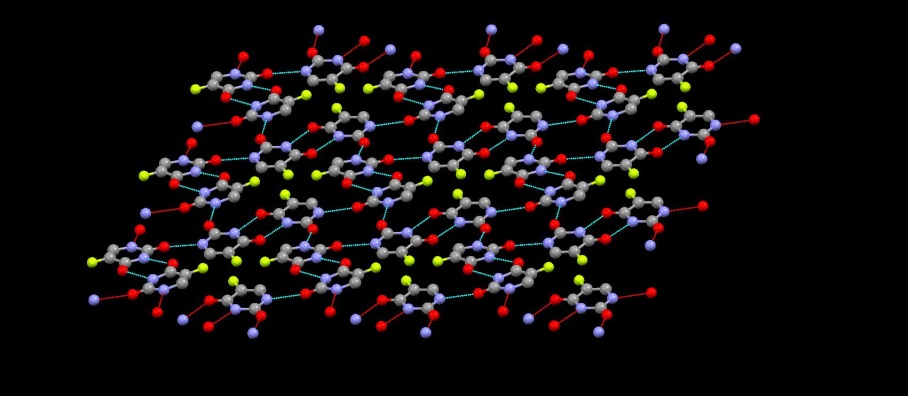


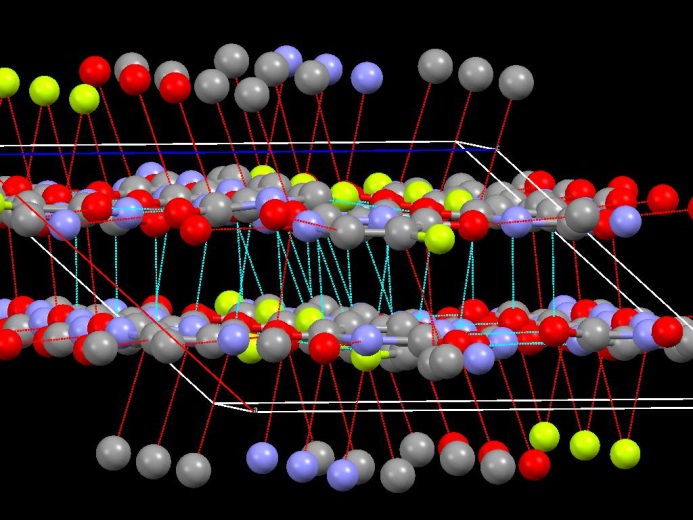


**Figure 2s.** The layering and hydrogen bonding in 5FU crystal [Crystallography Open Database

Information card for entry 5000078]

| **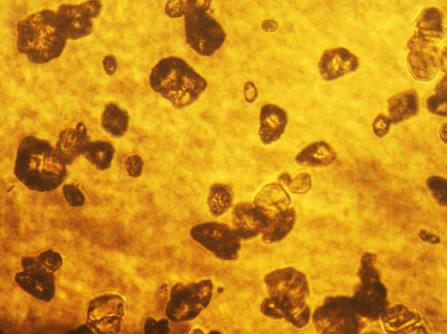** | **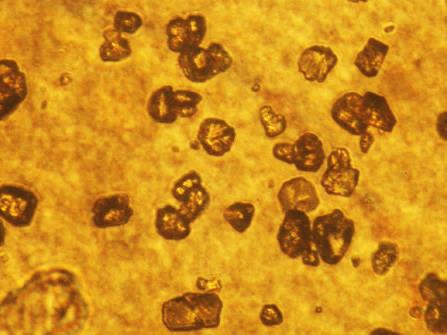** | **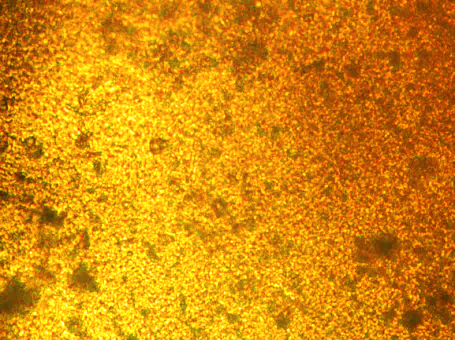** | **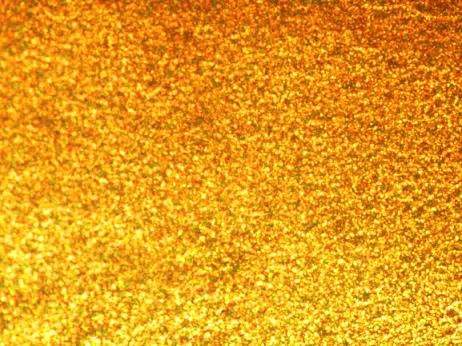** |
| --- | --- | --- | --- |
| **CFU1** | **CFU2** | **CFU3** | **CFU4** |

**Figure 3s.** Polarized optical microscopy images for the CFU hydrogels

**Figure 4s.** Calibration curve of 5FU drug in PBS

| **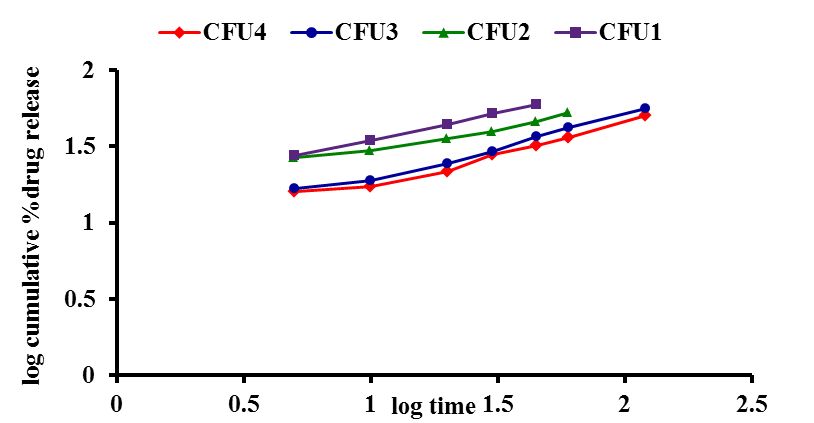a** | **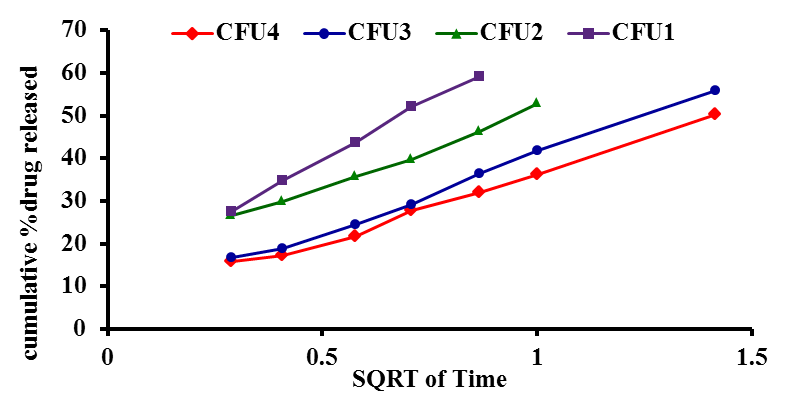**  **b** |
| --- | --- |

**Figure 5s.** Linear forms of Korsmeyer-Peppas (a) and Higuchi (b) models applied for the release

of 5FU from the CFU hydrogels

**Table 1s.** The parameters from the Korsmeyer-Peppas and Higuchi equations for the 5FU release from the CFU hydrogels

| **Sample** | **Korsmeyer Peppas** | | **Higuchi** | |
| --- | --- | --- | --- | --- |
|  | **R^2^** | **n_r_** | **R^2^** | **k_H_** |
| **CFU1** | 0.9986 | 0.4303 | 0.9976 | 45.402 |
| **CFU2** | 0.9729 | 0.4536 | 0.9915 | 32.724 |
| **CFU3** | 0.9792 | 0.4754 | 0.9946 | 32.24 |
| **CFU4** | 0.9688 | 0.422 | 0.9921 | 26.376 |

**Table 2s.** The influence of hydrogels administration on the OC, PC and BC levels.

Values were expressed as mean ± SD for 6 mice in a group.

|  | **OC (colonies/ml)** | **PC (colonies/ml)** | **BC (colonies/ml)** |
| --- | --- | --- | --- |
| **Distilled water** | **811.37±49.33** | **527.22±42.29** | **715.25±39.33** |
| **chitosan** | **797.29±36.45** | **529.47±32.44** | **706.58±32.75** |
| **5FU** | **794.51±45.67** | **522.51±39.57** | **709.35±44.29** |
| **CFU3** | **791.35±57.55** | **521.62±22.65** | **711.41±27.52** |
| **CFU4** | **790.43±39.31** | **524.37±41.34** | **707.29±37.43** |

**Acknowledgements**

The research leading to these results has received funding from the Romanian National Authority for Scientific Research, MEN-UEFISCDI grant, project number PN-III-P1-1.2-PCCDI2017-0569 and is part of a project that has received funding from the European Union’s Horizon 2020 research and innovation programme under grant agreement No 667387 WIDESPREAD 2-2014 SupraChem Lab.
